# Immunomodulatory properties of naïve and inflammation-informed dental pulp stem cell derived extracellular vesicles

**DOI:** 10.3389/fimmu.2024.1447536

**Published:** 2024-08-19

**Authors:** Sadiq Umar, Koushik Debnath, Kasey Leung, Chun-Chieh Huang, Yu Lu, Praveen Gajendrareddy, Sriram Ravindran

**Affiliations:** ^1^ Department of Oral Biology, University of Illinois, Chicago, IL, United States; ^2^ Department of Periodontics, University of Illinois, Chicago, IL, United States

**Keywords:** dental pulp stem cells, extracellular vesicles, inflammation, NFκB pathway, TNFα

## Abstract

Mesenchymal stem cell derived extracellular vesicles (MSC EVs) are paracrine modulators of macrophage function. Scientific research has primarily focused on the immunomodulatory and regenerative properties MSC EVs derived from bone marrow. The dental pulp is also a source for MSCs, and their anatomical location and evolutionary function has primed them to be potent immunomodulators. In this study, we demonstrate that extracellular vesicles derived from dental pulp stem cells (DPSC EVs) have pronounced immunomodulatory effect on primary macrophages by regulating the NFκb pathway. Notably, the anti-inflammatory activity of DPSC-EVs is enhanced following exposure to an inflammatory stimulus (LPS). These inhibitory effects were also observed *in vivo*. Sequencing of the naïve and LPS preconditioned DPSC-EVs and comparison with our published results from marrow MSC EVs revealed that Naïve and LPS preconditioned DPSC-EVs are enriched with anti-inflammatory miRNAs, particularly miR-320a-3p, which appears to be unique to DPSC-EVs and regulates the NFκb pathway. Overall, our findings highlight the immunomodulatory properties of DPSC-EVs and provide vital clues that can stimulate future research into miRNA-based EV engineering as well as therapeutic approaches to inflammation control and disease treatment.

## Introduction

Macrophages are key mediators of response to injuries and play an active role in the inflammatory stages of healing (1–7). These myeloid lineage derived cells, comprise a highly diverse population found throughout the body, from peripheral blood to various organs such as lungs, liver, brain, kidneys, and vascular endothelium (8). During fracture healing, macrophages produce various cytokines, including IL-1β, TNF-α, IL-6 and C-C chemokine ligand 2. IL-1β produced promotes angiogenesis and accelerate the growth of primary cartilaginous calluses. TNF-α is involved in cell recruitment and secondary inflammatory signaling. IL-6 can recruit mesenchymal stem cells (MSCs) to the injury site and stimulate osteoblast differentiation. Depending on the microenvironmental cues they receive, macrophages can undergo activation and develop distinct functional abilities: the proinflammatory (classically activated macrophages) and the anti-inflammatory (alternatively activated macrophages). Typically, macrophages display a neutral phenotype, which assists in maintaining tissue homeostasis. However, during the early phases of inflammation, they transition to an inflammatory phenotype (here in referred to as M1). These M1 macrophages generate proinflammatory cytokines and nitric oxide, leading to tissue damage. As inflammation resolves, macrophages predominantly transition towards a reparative phenotype (herein referred to as M2), that can suppress proinflammatory cytokine production, remove debris, and restore tissue equilibrium (9–11).

Among the signaling pathways regulating macrophage function, Toll-like receptors (TLRs) play a critical role by detecting various danger signals and by initiating inflammation by stimulating inflammatory cytokines (12). However, sustained activation is detrimental to tissue repair and/or regeneration (13–17). For example, when bacterial lipopolysaccharide (LPS), TLR ligands or other damage- associated molecular patterns (DAMPs) bind to TLR4, they activate Toll–IL-1 receptor (TIR) that is shared by both TLRs and interleukin-1 receptors (IL-1Rs). This interaction initiates a signaling cascade that leads to the recruitment of IL-1 receptor-associated kinases (IRAK1-4) by MyD88. TRAF6, acting as an E3 ubiquitin ligase, forms a complex with IRAK-1, which then dissociates from the receptor and translocate to the cytoplasm. In the cytoplasm, IRAK-1 recruits and activates TAK1 at its kinase domain threonine (Thr184/187), initiating downstream signaling cascades, including p38, JNK, and ERK, resulting in the production of proinflammatory cytokines such as TNFα, IL-1β, and IL-6, among others (18–22).

Recent research has shown that mesenchymal stem cells (MSCs) can reshape the immune response by modifying both innate and adaptive immunity through direct cell-cell communication or by releasing paracrine modulatory factors (1, 23–26). MSCs are derived from various tissues, including bone marrow, adipose tissue, and dental pulp (27). Dental pulp stem cells (DPSCs) have been reported to possess stronger immune-regulatory effect owing to their anatomical location in the dental pulp and its susceptibility to infection (28). Much of the activity of MSCs are mediated in a paracrine manner governed by MSC derived Extracellular vesicles (EVs). EVs are nano scale entities containing lipids, proteins and nucleic acids (mRNA, DNA and miRNA) that are secreted into the extracellular space by all cells under both normal and pathological conditions (24, 29, 30). EV uptake by recipient cells influences their behavior in homeostasis as well as response to various insults, injuries and disease. Multiple studies including ours, have demonstrated the anti-inflammatory and regenerative potential of MSC EVs (31–37) and their use as therapeutic agents in regenerative medicine.

While bone marrow derived MSC EVs have been studied extensively, relatively fewer studies have highlighted the potential of dental pulp stem cell derived EVs (DPSC EVs) from an immunological perspective. In a prior study, we have shown the potential of DPSC-EVs in stem cell differentiation (38). The primary focus of this study was to highlight the anti-inflammatory properties of DPSC-EVs with a secondary focus on identifying how the presence of an inflammatory environment will influence the DPSCs and subsequently, the properties of their EVs.

## Materials and methods

### Ethical statement

This study adheres to all pertinent ethical standards. All animal procedures were conducted in accordance with the guidelines set forth by the National Institutes of Health and the University of Illinois, Chicago’s Institutional Animal Care and Use Committee. The animal study was reviewed and approved by University of Illinois Chicago Animal Care Committee (**Protocol No.:** 23-184).

### Cell culture

Human DPSCs were procured from Lonza (Catalog: PT-5025) and cultured in alpha-MEM containing 20% fetal bovine serum (Gibco), 1% L-Glutamine (Gibco) and 1% antibiotic-antimycotic solution (Gibco). Mouse bone marrow derived macrophages (mBMMs) were isolated from 8-week C57BL/6J mice. Mouse bone marrow cells were cultured with mouse M-CSF (20 ng/ml) for 5-7 days to obtain myeloid cells differentiated *in vitro* as MΦs (10% FBS/DMEM). For polarizing macrophage in to M1 or M2, mBMMs were stimulated with lipopolysaccharides (100 ng/ml, Sigma) with Interferon gamma (50 ng/ml, Peprotech) or Interleukin 4 (20 ng/ml, Peprotech) for 24 hours (20, 31).

### EV isolation and characterization

EVs were isolated from the naïve and LPS preconditioned DSPCs according to established protocols (39, 40). Briefly, DPSCs were preconditioned with PBS or LPS (100 ng/ml) for 72 hours and cells were washed in serum free medium and cultured under serum free condition for 48 hours. The culture medium was harvested and centrifuged (1500 ×*g)* for 15 min to remove of cell debris. The medium was used to isolate EVs as per our established protocols using PEG-based isolation reagent (4x). Briefly, the conditioned medium was incubated with the reagent such that the final concentration of the reagent was 1x at 4°C overnight. Following this, the mixture was centrifuged in a refrigerated tabletop centrifuge for 30minutes at 1500g. The pellet containing the EVs was then resuspended in phosphate buffered saline (PBS) as per the requirements of the experiment.

### Characterization of EVs

EVs were isolated from serum-free media using the ExoQuick TC isolation reagent (System Biosciences). Nanoparticle tracking analysis (NTA) with a NanoSight NS300 instrument (Malvern Instruments, MA, USA) was employed to determine the size distribution and concentration of EV samples. For the assessment of exosomal markers, EVs were resuspended in RIPA buffer and equal lysates were loaded onto precast 4–20% polyacrylamide Mini-PROTEAN TGX gels (Bio-Rad Laboratories, Hercules, CA, USA). The presence of EV markers CD63 (ab59479, Abcam) and TSG101 (ab125011, Abcam) was examined. Transmission electron microscopy (TEM) was performed to validate the presence of exosomes in isolated samples. EV preparations (5 μL) were fixed in 2% paraformaldehyde (PFA) and applied onto formvar carbon-coated electron microscopy grids. Following incubation for 30 min at room temperature in a dry environment, the EV preparations were fixed in 1% glutaraldehyde for 5 min, washed in distilled water (8 water washes, 2 min each), contrasted in 2% uranyl-oxalate for 5 min, and embedded in a mixture of 4% uranyl-acetate and 2% methyl cellulose for 10 min on ice. The EVs were observed and imaged using a JEOL JEM 1011 TEM (JEOL Ltd., Tokyo, Japan).

### Quantitative and qualitative endocytosis of EVs

For quantitative endocytosis experiments, EVs were fluorescently labeled using CellTracker™ Green CMFDA (C7025, Thermofisher Scientific). mBMMs cells were plated in 96 well plates at a density of 10,000 cells per well. The cells were then incubated with increasing amounts of fluorescently labeled EVs for 2 h at 37°C resuspended in PBS. Sham labeled PBS alone was used as a control and the data were normalized to this control. Cells were subsequently washed with PBS and fluorescence was measured using a BioTek Cytation plate reader. For qualitative endocytosis experiments, 50,000 mBMMs were seeded onto cover glasses placed in 12 well cell culture plates. Fluorescently labeled EVs (1.8 x10^9^) particles/well) were added and incubated for 2 hours. The cells were then washed with PBS, fixed in 4% PFA, permeabilized and counter stained using Alexa Fluor^®^ 568 Phalloidin (1/2000, A12390, Invitrogen) antibody. The cover glasses were then mounted using mounting medium with DAPI (Vector Laboratories) and imaged using a Zeiss LSM 710 Meta confocal microscope.

### ELISA for cytokine measurements

Mouse *invitro differentiated* macrophages (mBMMs) were pretreated with EVs from Naïve or LPS preconditioned DPSC with LPS/IFN-γ or IL-4 for 24 hours. Conditioned media was collected and cytokine levels of IL-1β, IL-6, TNF-α, IL-10, and TGF-β were measured using DuoSet ELISA (enzyme-linked immunosorbent assay) kits (R&D Systems, MN).

### Real-time RT-PCR

RNA was extracted using the RNeasy^®^ Mini Kit (Qiagen) and then reverse transcribed to cDNA using the RevertAid RT Reverse Transcription Kit (Thermo Scientific) for subsequent quantitative real-time PCR (qRT-PCR) analysis. qRT-PCR was conducted using mouse primers obtained from Integrated DNA Technologies for iNOS, Interleukin-1 beta (IL-1β), Tumor Necrosis Factor alpha (TNF-α), Interleukin-6 (IL-6), Arginase 1 (Arg1), Interleukin-10 (IL-10), CD206, and YM-1, along with the SYBR green gene expression master mix (Applied Biosystems). The data are presented as fold changes in RNA levels compared to the control treatment, calculated using the 2−ΔΔCt method.

### Western blot analysis

Mouse bone marrow macrophages (mBMMs) were pretreated overnight with EVs from naive and LPS preconditioned DPSC. Cells were stimulated with LPS/IFN-γ for 30 min. Cells were washed with PBS and lysed in RIPA buffer containing protease and phosphatase inhibitors. Lysates were examined for TRAF6 (67591S), p-p38 (4511S), p-JNK (9251S), p-ERK (4370S) and p- NFκb (3033S) (1:1000, Cell Signaling) and GAPDH (2118S) for equal loading (1:3000, Cell Signaling).

### miRNA sequencing analysis

RNA isolation was performed using miRNeasy Mini Kit (Qiagen) as per the manufacturer’s protocol. Libraries were constructed using 500 ng of total RNA from EVs from Naïve and LPS preconditioned DPSC cells using TruSeq Small RNA Sample Prep Kit (Illumina). Libraries were multiplexed and sequenced on a HiSeq 2500 using TruSeq Rapid SBS sequencing chemistry v2 at the UIC Core Genomics Facility. Fastq files were generated with the bclfastq v1.88.4 and adapter sequences and low-quality sequences were removed, and miRNAs were identified with miRbase. The heatmap of top 25 miRNAs was generated based on multi-group statistic over all groups, using a fixed dispersion in edgeR to compute p-values.

### Rat calvarial defect model

To evaluate the effects of EVs on bone healing, the rat calvaria defect model was used (41). Each experimental group included 8 defects. The rats were anesthetized intraperitoneally using Ketamine (80 mg/kg)/Xylazine (10 mg/kg) and a vertical incision was made in the head at the midline to expose the calvarial bone. Two 5mm calvarial defects were created bilaterally in the calvarium without dura perforation using a trephine burr. A clinical grade collagen sponge (OraPLUG, Salvin) was placed on the wound with PBS (control), control EVs or LPS EVs (approximately 4x10^9^) particles/defect). The rats were sacrificed by carbon dioxide asphyxiation followed by cervical dislocation at each timepoint. For early time points, the embedded scaffolds were harvested and subjected to immunohistochemistry at day 1, 3 and 5 post-surgeries.

### Immunohistochemistry

The calvaria samples were decalcified in 10% EDTA solution. The harvested scaffolds at day 1, 3, and 5 and the decalcified calvaria were then embedded in paraffin and sectioned into 10 μm sections. For immunofluorescent staining, the slides were incubated in 5% BSA blocking buffer for an hour at room temperature. Macrophage markers were stained with rabbit polyclonal anti-inducible nitric oxide synthase (iNOS) antibody (1/100, ab15323, Abcam), rabbit monoclonal [D4E3M] anti-arginase 1 (Arg1) antibody (1/100, 93668, Cell signaling), and TNF-α (1/100, 11948S, Cell signaling), TRAF6 (1/100, 11948S, Cell signaling). Sections were then stained with anti-mouse FITC and anti-rabbit TRITC secondary antibodies (1/200, Sigma) and imaged using Zeiss LSM 710 laser scanning confocal microscope. ImageJ was used to quantify the immunostained number or %area per field (n=4 per group). The positive number of iNOS, Arg1, TNF-α and TRAF6 cells was normalized to the number of nuclei per field.

### Transfection of miRNA mimics and antagomirs

Mouse *invitro differentiated* macrophages (mBMMs) were seeded on to 24 well plate with m-CSF (20 ng/ml) for 5-7 days. Cells were transfected with miR-320a-3p mimic (Dharmacon) at a final concentration of 100 pmol using Lipofectamine 3000 (Thermo Fisher) complying with the manufacturer’s instruction. The transfected cells were utilized following 48h of transfection and were either untreated or stimulated with LPS/IFN-γ with or without EVs from Naive DPSC for 24h. Total RNA was isolated and subjected to RT-PCR. Data are presented as fold changes in RNA levels compared to control treatment, calculated following the 2−ΔΔCt method.

### Statistical analysis

The normal distribution of the data obtained from the experiments was evaluated using the Shapiro-Wilk test. For the experiments involving comparison of more than two groups, one-way ANOVA was performed with P<0.05 as confidence interval. Pairwise comparisons were performed using Tukey’s/Šídák’s *ad-hoc* test with a confidence interval of 95% (P<0.05).

## Results

### Dental pulp stem cells exhibit superior anti-inflammatory activity compared to human bone marrow derived mesenchymal stem cells

**Figure 1:**
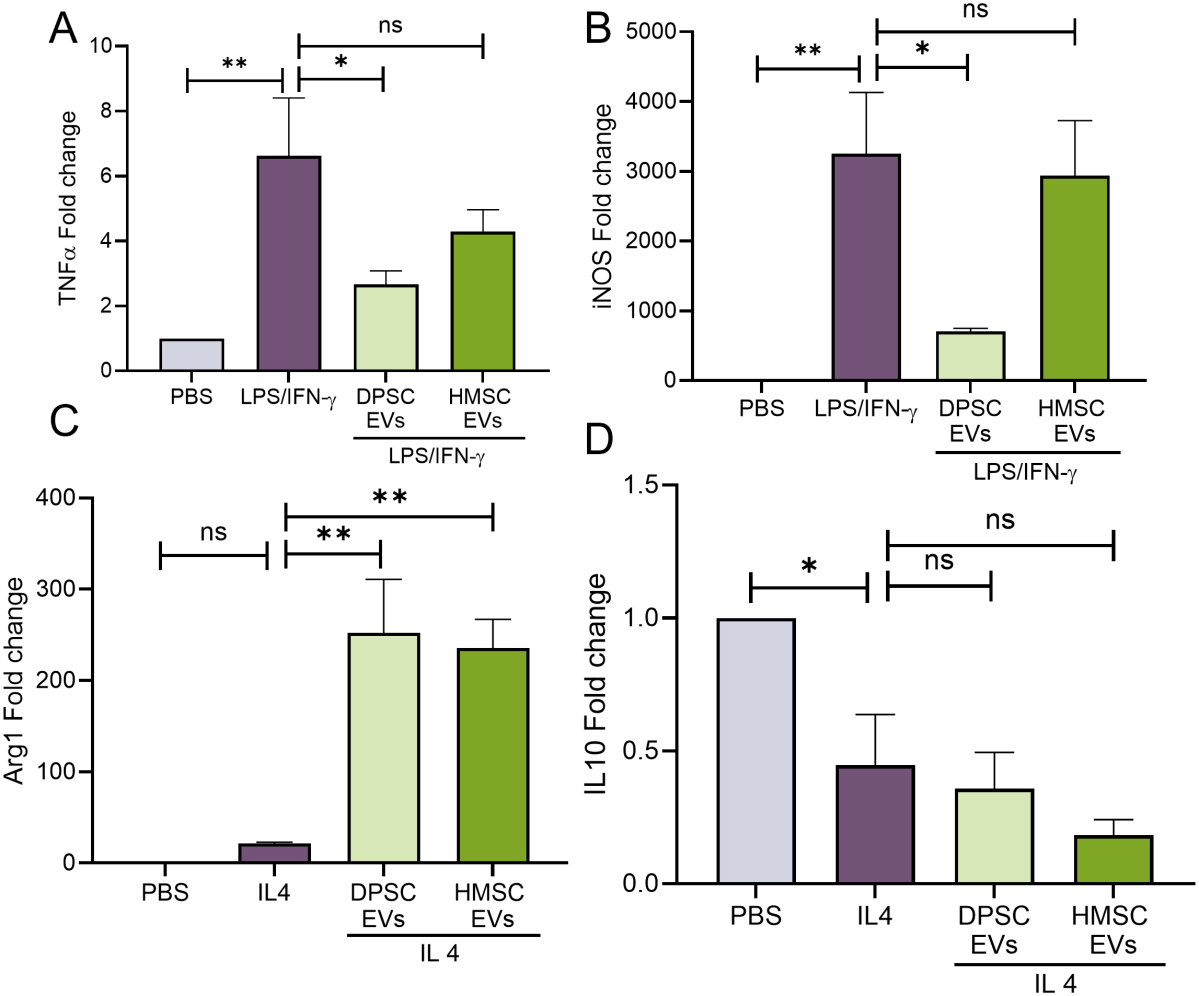
EVs from DPSC strongly reverses macrophage polarization than HMSC. **(A–D)** mouse bone marrow macrophages (mBMMΦs) were pretreated with EVs from DPSC or hBMSC with PBS or LPS/IFNγ (100 and 50 ng/ml) or IL4 (20ng/ml) for 24hr and analyzing the gene levels of TNF-α **(A)**, iNOS **(B)**, Arg1 **(C)** and IL10 **(D)** by real-time RT-PCR, n=3. The data are shown as mean ± SEM, * represents p<0.05 and ** denotes p<0.01. Significant differences were determined by one-way ANOVA following Šídák’s multiple comparison test. ns, not significant.

We and others have shown the anti-inflammatory properties of bone marrow derived MSC EVs. Specifically, treatment with naïve hBMSC-EVs significantly reduces IL1β expression. However, TNFα and iNOS expressions are minimally altered (31). Here, we compared the anti-inflammatory activity of bone marrow and DPSC derived EVs *in vitro* on primary bone marrow macrophages (mBMMΦs) with respect to iNOS and TNFα; expression. Inflammatory polarization (M1-like) of BMMΦs with LPS/IFN-γ resulted significant increases in the gene expression of cytokines TNF-α and iNOS (**Figures 1A, B**). Results indicated that DPSC-EVs significantly attenuated this increase while hBMSC-EVs did not show any statistically significant change with respect to control polarized mBMMΦs (**Figures 1A, B**). We next polarized the macrophages to a reparative phenotype (M2-like) by stimulation with IL4. This resulted in an increased expression of Arg1, a reparative macrophage phenotypic marker. In the presence of the EVs, Arg1 expression was enhanced even more and no significant trends or changes were observed between DPSC and hBMSC-EVs treated groups (**Figures 1C, D**). Overall, these results suggest that extracellular vesicles isolated from DPSCs possess both anti-inflammatory and pro-reparative activity towards mBMMΦs and that the activity of DPSC-EVs with respect to iNOS and TNFα differs from that of hBMSC-EVs.

### LPS pre-conditioned DPSC-EVs showed enhanced anti-inflammatory activity compared to naïve DPSC EVs

**Figure 2:**
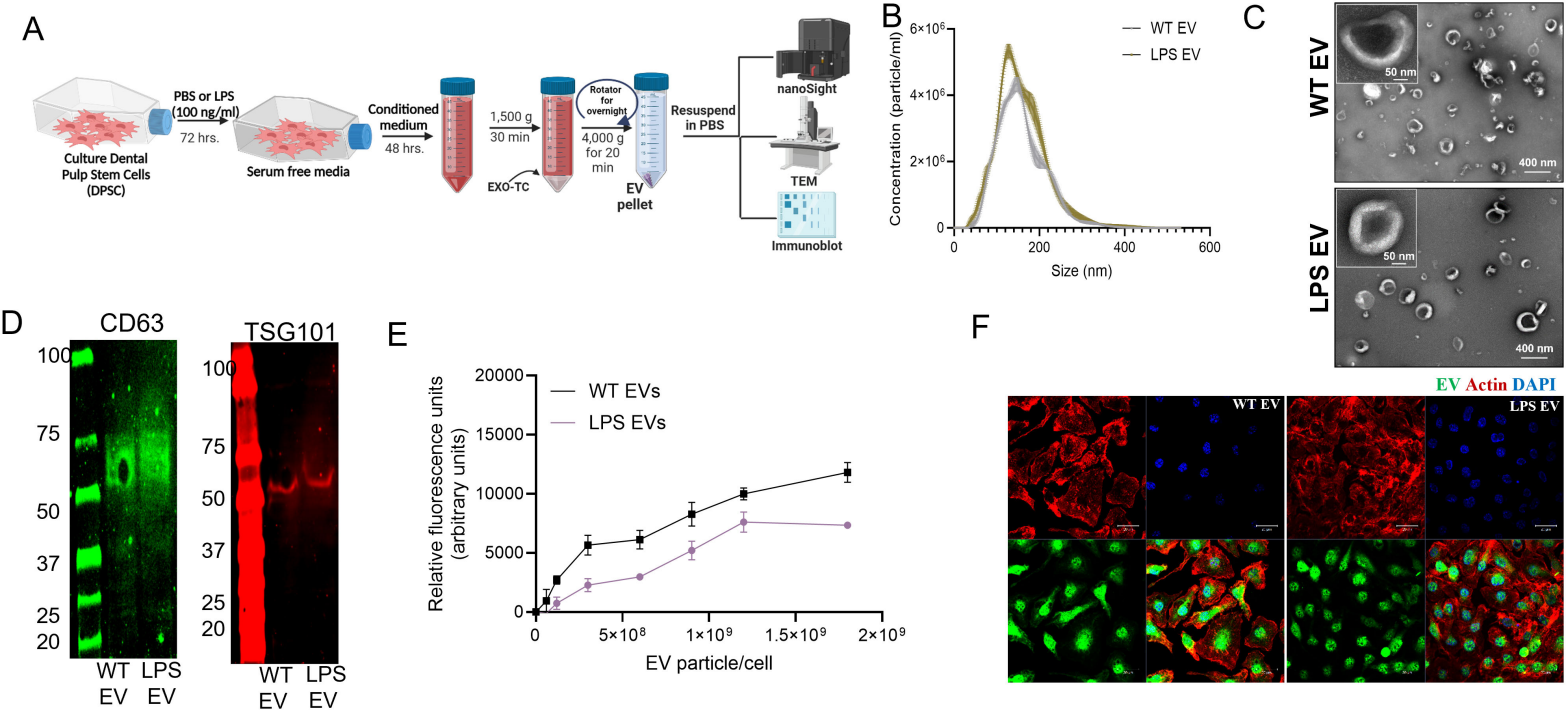
EVs from naïve and LPS pre-conditioned DPSCs showed similar EV characteristics. **(A)** schematic diagram to show the stimulation and isolation of EVs from regular (WT EVs) and LPS preconditioned DPSC (LPS EVs) **(B)** NTA plots of WT and LPS EVs showing their size distribution and **(C)** representative image of EVs as observed by transmission electron microscopy (TEM) **(D)** expression of EV markers CD63, and TSG101. **(E)** mBMMΦs were treated with fluorescently labeled WT and LPS EVs dose dependently and incubated for 2hr. Data represents mean ± SEM (*n* = 6). **(F)** Representative confocal images of fluorescently labeled EVs (green) endocytosed by mBMMΦs, mBMMΦs were counterstained with actin (red) and the nuclei were stained with DAPI (blue).

Our studies with hBMSC-EVs have shown that stimulation or preconditioning of stem cells with inflammatory stimulus or cytokines resulted in the cells generating EVs with enhanced immunomodulatory activity. These inflammatory pre-conditioned EVs showed an ability to attenuate iNOS and TNFα expression. Here, we investigated if DPSC EV properties are altered when parental cells are subjected to inflammatory stimulus (LPS). DPSCs were preconditioned with LPS and EVs were isolated as per established protocols. We began by characterizing the basic properties of naïve and inflammation preconditioned DPSC EVs. Results presented in **Figure 2** shows that inflammatory preconditioning does not alter the particle size (measured by NTA), morphology (evaluated by TEM) and EV marker expression (determined by immunoblotting). Quantitative and qualitative endocytosis experiments were then performed to evaluate the endocytic ability of the EVs by mBMMΦs. Results presented in **Figures 2E, F** show that both the naïve and LPS preconditioned DPSC-EVs showed similar ability to be endocytosed.

**Figure 3:**
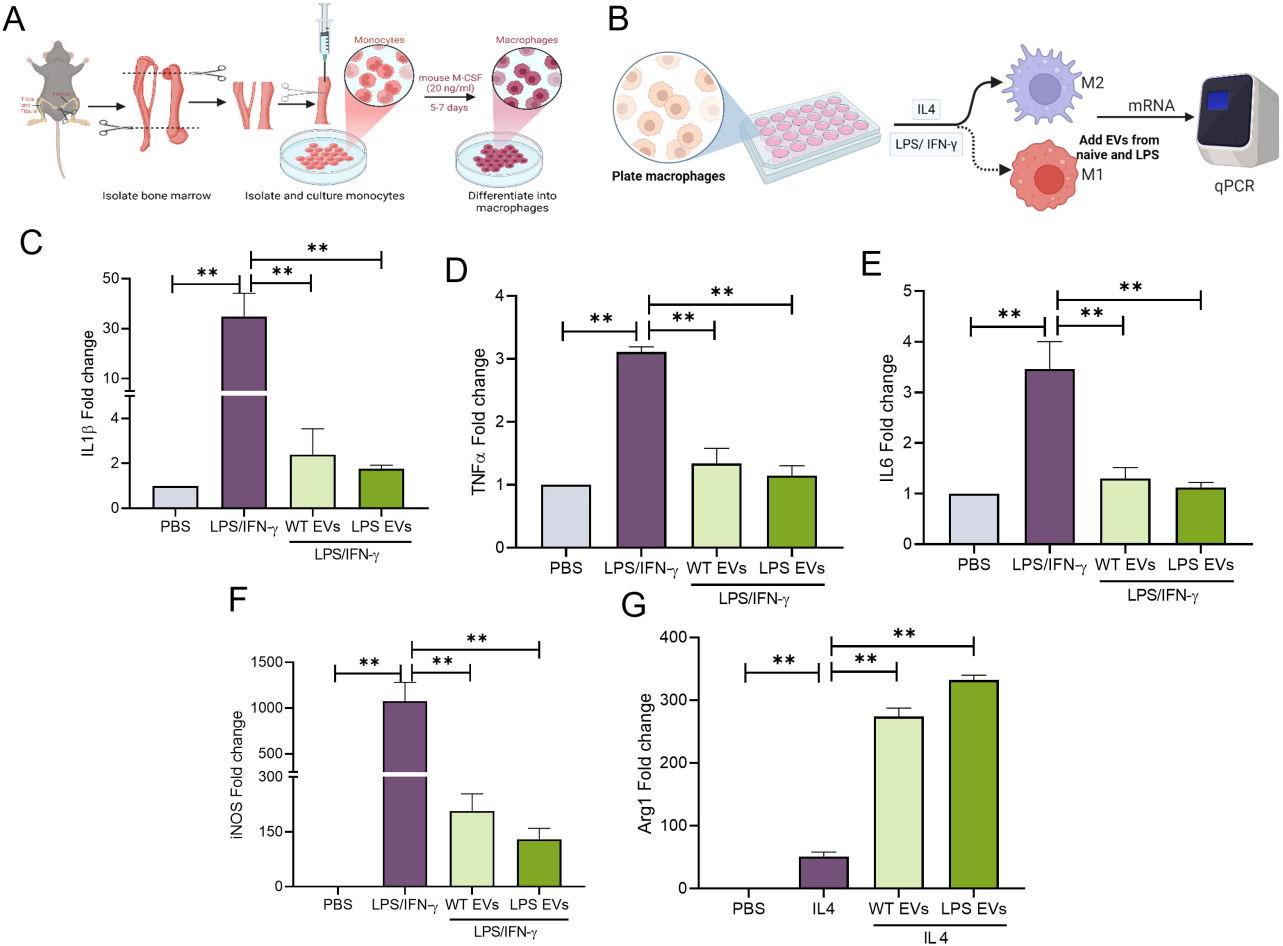
LPS pre-conditioned DPSC-EVs showed enhanced anti-inflammatory activity. **(A)** schematic diagram to show isolation of monocytes from bone marrow and its differential into macrophages by mouse m-CSF, also **(B)** experimental conditioned and time to isolate RNA. **(C–G)** mBMMΦ were stimulated with LPS/IFN-γ (100/50 ng/ml) or IL4 (50 ng/ml) for 24 hr with or without EVs from WT and LPS preconditioned DPSC. Cell was harvested for RNA isolation and mRNA expression of IL-1β, TNF-α, IL6, iNOS and arg1 by real-time RT-PCR, n=3. The data are shown as mean ± SEM, ** denotes p<0.01. Significant differences were determined by one-way ANOVA following Šídák’s multiple comparison test. ns, not significant.

**Figure 4:**
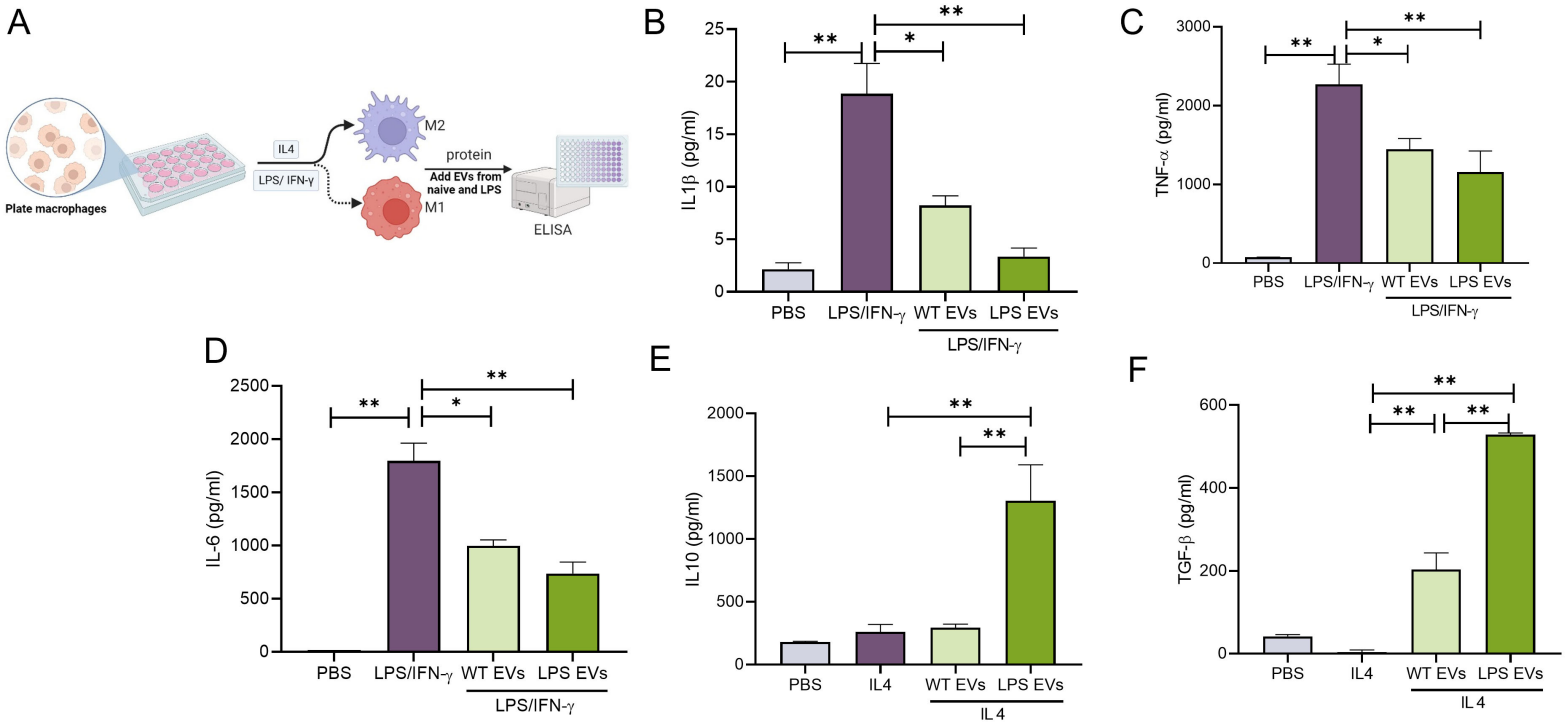
LPS pre-conditioned DPSC-EVs displayed greater immunomodulation at protein level. **(A)** schematic diagram to show isolation of monocytes from bone marrow and its differential into macrophages by mouse m-CSF, also **(B)** experimental design to collect conditioned media. mBMMΦ were stimulated with LPS/IFN-γ (100/50 ng/ml) or IL4 (50ng/ml) for 24 hr with or without EVs from WT and LPS preconditioned DPSC. Conditioned media was utilized for quantifying cytokines such as IL-1β, TNF-α, IL-6, IL10 and TGF-β **(B–F)** secretion by ELISA. n=3. The data are shown as mean ± SEM, * represents p<0.05 and ** denotes p<0.01. Significant differences were determined by one-way ANOVA following Šídák’s multiple comparison test.

To evaluate the immunomodulatory properties of the EVs, mBMMΦs were polarized towards M1 or M2 phenotypes. M1 polarization was induced with LPS/IFN-γ, while M2 polarization was induced with IL-4. Results presented in **Figure 3** (gene expression) and **Figure 4** (protein expression) demonstrate that in mBMMΦs, LPS-preconditioned EVs inhibited the expression of M1-associated genes and proteins while enhancing M2-associated ones. Specifically, mRNA expression levels of M1 genes IL-1β (34-fold), TNF-α (3-fold), IL-6 (4-fold), and iNOS (1100-fold) were significantly increased by LPS/IFN-γ, whereas M2 genes IL-10 (0.5-fold) and ARG1 (51-fold) were upregulated by IL-4. LPS-preconditioned EVs exhibited greater potency in abolishing M1 polarization and enhancing M2 polarization compared to naïve EVs. Specifically, IL-1β, TNF-α, and IL-6 were significantly reduced (60-80%) by LPS-preconditioned EVs compared to naïve EVs (35-55%). Additionally, a remarkable increase in IL-10 (500%) and TGF-β (16000%) secretion was observed in LPS-preconditioned EVs compared to naïve EVs, indicating enhanced polarization of macrophages towards the reparative phenotype upon EV treatment. Overall, LPS-preconditioned EVs were marginally more effective than naïve DPSC-EVs in controlling macrophage polarization by inhibiting inflammatory polarization and significantly more effective in enhancing reparative phenotype polarization.

### Extracellular vesicles preconditioned with LPS disrupt TLR4 signaling

**Figure 5:**
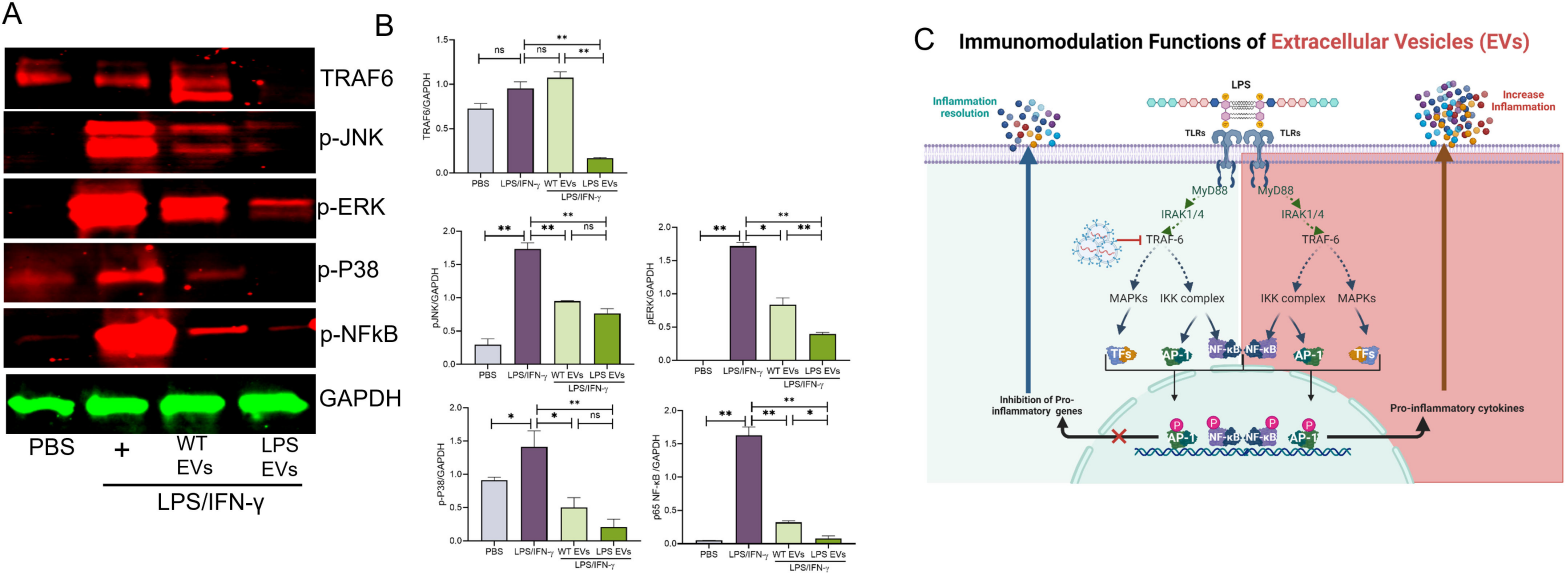
Pre-conditioned DPSC-EVs possessed enhanced activity in suppressing the NFκb pathway mBMMΦ were seeded in to 6 well plate and incubated overnight with EVs with WT and LPS preconditioned DPSC and stimulated with LPS/IFN-γ (100/50 ng/ml) for 30 min. Lysates were probed for p-ERK, p-p38, p-JNK, TRAF6 and NFκb (p65) (1:1000, Cell signaling) and normalized to GAPDH (1:3000, Cell signaling), n=3. Representative images shown in **(A)**. Western blot density was analyzed by Image J **(B)**. The data are shown as mean ± SEM, * represents p<0.05 and ** denotes p<0.01. Significant differences were determined by one-way ANOVA following Šídák’s multiple comparison test. **(C)**. Schematic illustration showing the possible mechanism of EVs in TLR signaling. ns, not significant.

Based on the activity of DPSC-EVs with respect to TNFα and iNOS, we delved into the ability of LPS-preconditioned extracellular vesicles (EVs) to modulate Toll-like receptor 4 (TLR4) signaling in mBMMΦs. Activation of Toll-like receptor 4 (TLR4) by lipopolysaccharide (LPS) triggers the release of key proinflammatory cytokines essential for initiating robust immune responses. We observed that stimulation with LPS/IFN-γ led to the phosphorylation of P38, JNK, ERK, and NFκb along with activation of TNF-α expression. Remarkably, these activations were completely abolished by LPS-preconditioned EVs, whereas naïve EVs exhibited significant inhibition of these pathways (**Figure 5**). Specifically, LPS-preconditioned EVs significantly inhibited the phosphorylation of P38, JNK, ERK, and NFκb induced by LPS/IFN-γ, effectively dampening downstream proinflammatory signaling cascades. Additionally, the activation of TNF-α expression, a key proinflammatory cytokine, was markedly suppressed by LPS-preconditioned EV compared to naïve DPSC EV effects. While some inhibition was observed with naïve EVs, it was notably less potent compared to the effects of LPS-preconditioned EVs. This suggests that LPS preconditioning of DPSCs confers a superior ability to its derivative EVs to modulate TLR4 signaling and mitigate proinflammatory responses in mBMMΦs.

### *In vivo* effects of naïve and LPS preconditioned DPSC-EVs

**Figure 6:**
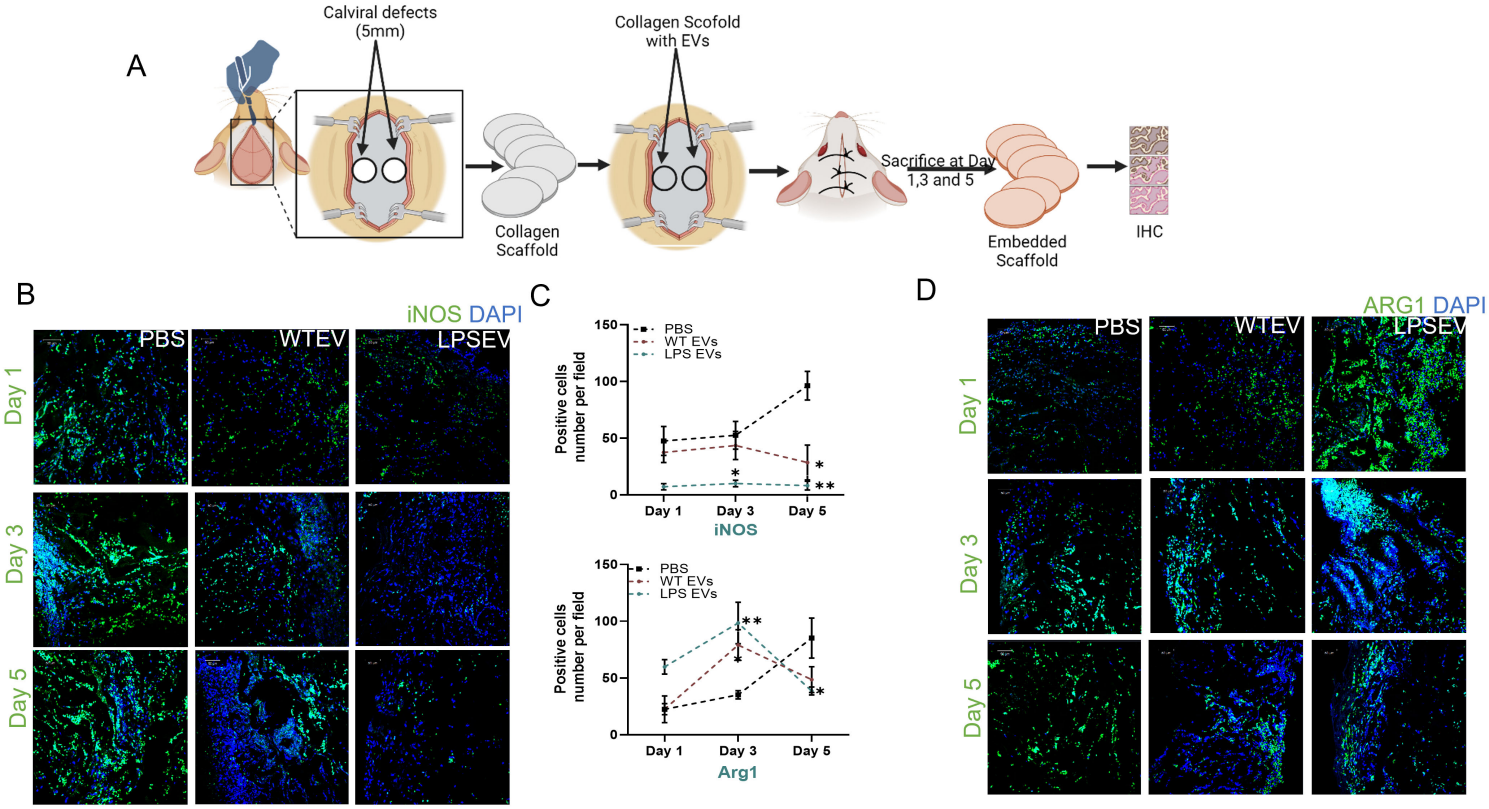
*In Vivo* inflammatory response is modulated by LPS preconditioned EVs. **(A)** schematic diagram to experimental design and harvesting of samples at day 1,3 and 5. iNOS (M1) and ARG1(M2) expression in rat calviral defect model **(B)** Representative confocal micrographs of 1-, 3- and 5-day calvarial sections immunoassayed for iNOS or **(C)** Quantification of positive cells are presented as mean ± SEM, * represents p<0.05 and ** denotes p<0.01. Significant differences were determined by one-way ANOVA following Šídák’s multiple comparison test. **(D)** ARG1 (green) and nuclear staining by DAPI (blue).

**Figure 7:**
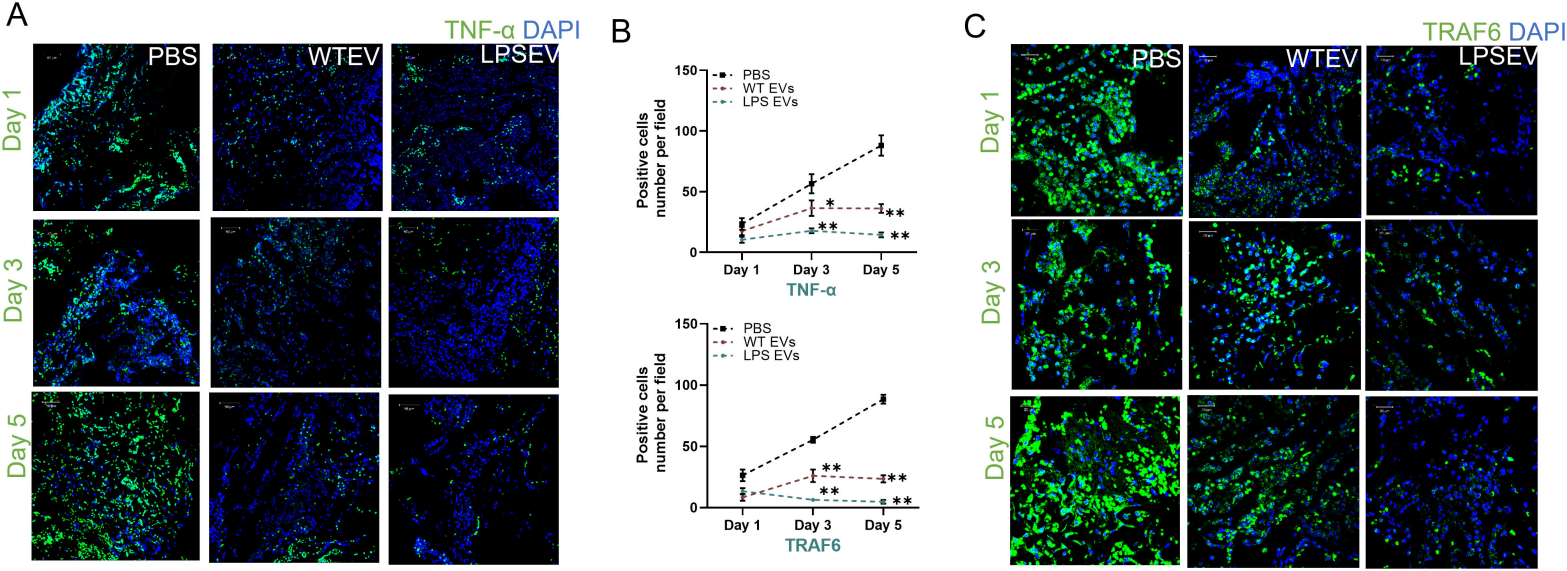
LPS preconditioned EVs regulate inflammatory mediator in rat calviral model. Representative confocal micrographs of 1-, 3- and 5-day calvarial sections immunoassayed for TNF-α **(A, B)** Quantification of positive cells are presented as mean ± SEM, * represents p<0.05 and ** denotes p<0.01. Significant differences were determined by one-way ANOVA following Šídák’s multiple comparison test. **(C)** or TRAF6 (green) and nuclear staining by DAPI (blue).

Our *in vitro* results demonstrate that LPS preconditioning can decrease pro-inflammatory activity and boost the anti-inflammatory/reparative functions of macrophages. To validate these findings *in vivo*, we employed a rat calvarial defect model to assess the impact of naïve and LPS preconditioned extracellular vesicles (EVs) on inflammation sustained from injury. We examined the effects of EVs qualitatively and quantitatively at days 1, 3, and 5 post-wounding. The wounds were treated with collagen membranes containing either the respective EVs or PBS, and samples were collected at the specified time points. Using immunohistochemistry on paraffin-embedded samples, we assessed iNOS as a pro-inflammatory marker (M1-like) and ARG1 as an anti-inflammatory marker (M2-like) followed by immunohistochemistry for TNFα and TRAF6 as representatives of the NDκb pathway. The confocal images presented in **Figures 6** and **7** are representative images from the paraffin embedded sections and the areas to be imaged were randomly selected to avoid bias. Our results indicate a reduction in the number of iNOS positive cells in the LPS EV group compared to untreated (PBS) and naïve EV group at all time points suggesting a shift towards resolution of inflammation (**Figures 6B, C**). ARG1 levels were higher in the EV treated groups at days 1 and 3 but control group showed increased presence of ARG1 positive cells at day 5 (**Figures 6C, D**). TRAF6 and TNFα IHC indicated that LPS EVs effectively abolished their expression, significantly more so than PBS or naïve EVs (**Figures 7A–C**). Overall, these findings support our *in vitro* observations, indicating that LPS preconditioning enhances the immunomodulatory effects of DSPC EVs by reducing the expression of pro-inflammatory markers and enhancing the expression of reparative anti-inflammatory markers.

### miRNA profile of LPS-preconditioned extracellular vesicles

**Figure 8:**
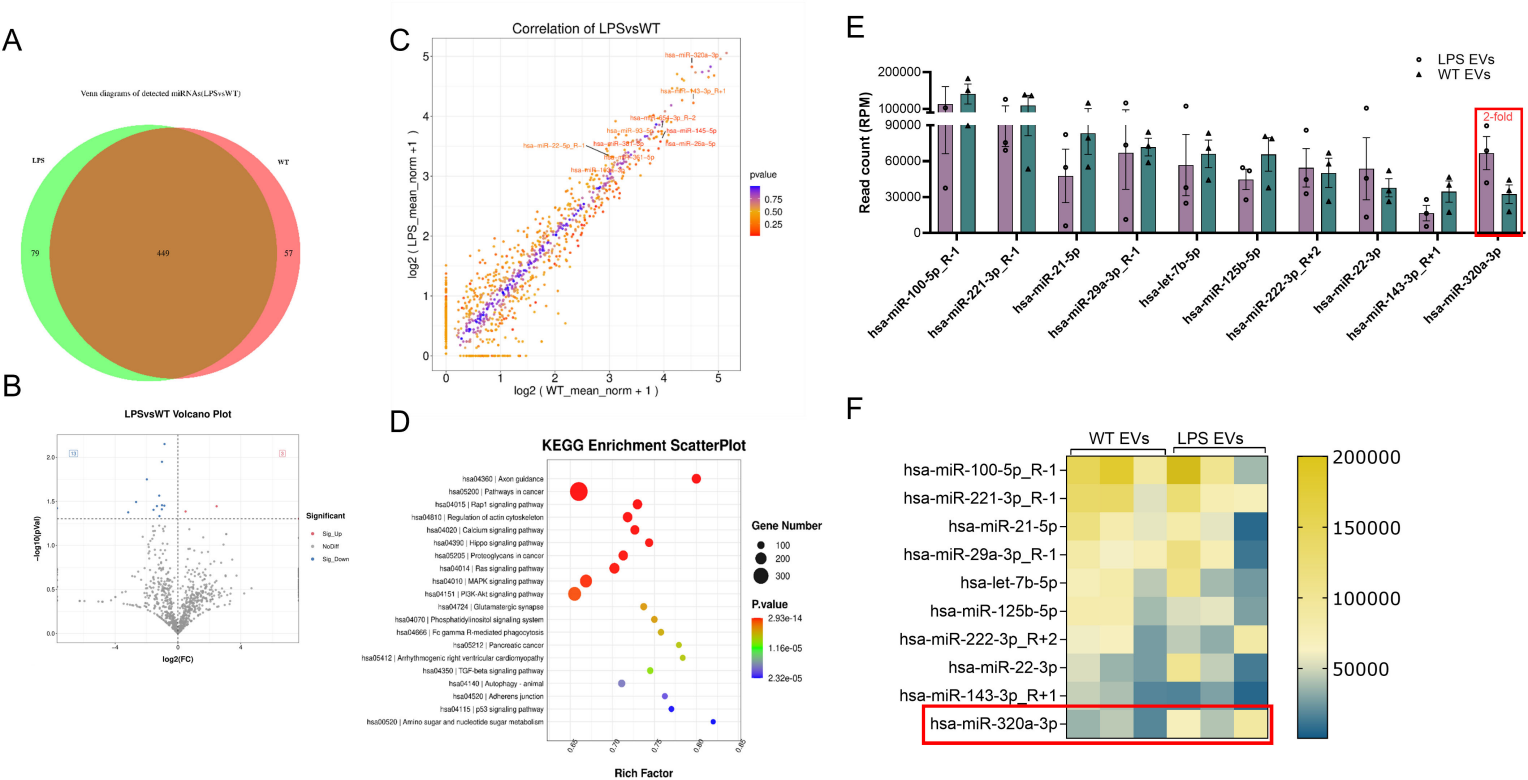
miR-Seq analyses revealed significant changes to EV miRNA composition. DPSC were preconditioned with PBS or LPS (100 ng/ml) for 72 hr and cells were washed with PBS and further incubated with serum free media for 48 hr. Total mRNA was isolated from EVs and sequenced. miRNA composition in WT and LPS preconditioned EV were shown **(A)** Venn diagram **(B, C)** Volcano plot of differentially expressed miRNAs in naïve and LPS EVs isolated from DPSC **(D)** KEGG analysis of relevant pathways significantly affected by change in miRNA composition of EVs. **(E, F)** EVs from DPSC preconditioned with and without LPS and shown top 10 miRNAs by bar chart and heatmap (n = 3).

Our prior studies have highlighted the role of EV miRNA cargo on EV function (31, 42). Here, we sought to identify the miRNA cargo of DPSC-EVs and evaluate how this might be altered by inflammatory preconditioning. To accomplish this, we stimulated DPSC with or without LPS and isolated the EVs, as described previously, and preformed miRNA sequencing. Comparative analysis with EVs from unstimulated DPSC revealed differential expression of 3 upregulated and 13 significantly downregulated miRNAs in LPS-treated EVs (**Figure 8**). To pinpoint miRNAs that may be involved in regulating the TRAF6 expression and downregulating NFκb signaling pathway, we utilized online prediction tools to compile a list of shared miRNAs identified in miRDB. Here, we limited our criteria to top 10 most expressed miRNAs in the EVs as they constitute more than 70% of the total EV miRNA composition. We then compared this list to that of the top 10 EV miRNA from hBMSC-EVs from our prior published study (**Supplementary Figure 1**). We further compared preconditioned DPSC-EVs with and without LPS and identified our top candidate as miR-320a-3p which increases 2-fold (that is absent in hBMSC-EVs cargo) and specifically targets the NFκb pathway (YOD1-TRAF6). miRDB target expression analysis of miR320a-3p showed a target score of 100 for YOD1 which directly associates with TRAF6 and competes with p62 for binding and activation of TRAF6. The reduction of YOD1 results in an increased activation of canonical NFκb. In summary, our results suggest that LPS-treated EVs might modulate the TLR4/NF-κB signaling pathway via miR-320a-3p, thereby ameliorating pathology and inflammatory responses.

### LPS induced inflammation abrogated by miR-320a-3p mimic in macrophages

**Figure 9:**
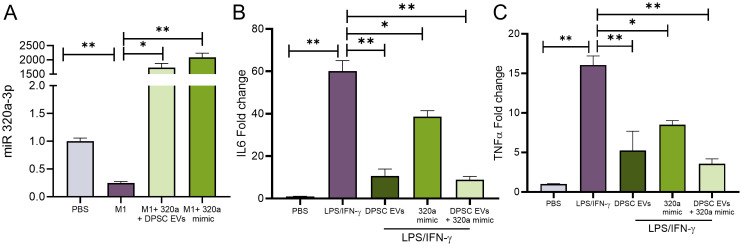
miR-320a-3p Function in regulation of inflammation. mBMMΦ were transfected with miR-320a-3p at a final concentration of 100 pmol using Lipofectamine 3000 (Thermo Fisher) following the manufacturer’s instruction. The transfected cells were utilized following 48h of transfection and were either untreated or stimulated with LPS/IFN-γ. mRNA harvested from these cells were used to analyze **(A)** expression of miR-320-3p and cytokines levels of IL6 and TNF-α **(B, C)** by real-time RT-PCR, n=3. The data are shown as mean ± SEM, * represents p<0.05 and ** denotes p<0.01. Significant differences were determined by one-way ANOVA following Šídák’s multiple comparison test.

Predictions indicate that miR-320a-3p potentially targets TRAF6, a crucial mediator within the NF-κB signaling pathway. Certain miRNAs possess the capability to bind to the 3’ untranslated region (UTR) of YOD1, a known partner of TRAF6. To verify the regulatory role of miR-320a-3p on inflammation in macrophages, mBMMΦs were transfected with miR-320a-3p mimics. Subsequently, cells were exposed to LPS/IFN-γ with or without naïve DPSC-EVs and the mRNA levels of inflammatory genes were assessed. We observed a significant decrease in IL-6 and TNF-α levels with the mimic, which were further reduced when treated in conjunction with naïve DPSC-EVs (**Figure 9**). Additionally, we noted an increase in miR-320a-3p expression in cells treated with the mimic and naïve EVs, although there was a decrease in expression with LPS/IFN-γ treatment. This indicates that during inflammation, miR-320a-3p expression maybe reduced, but restoring its expression can aid in mitigating inflammation.

## Discussion

Several studies over the past decade, including ours, have highlighted the anti-inflammatory properties of MSC EVs (31, 32, 40, 43, 44). However, the majority of the scientific work is focused on studying bone marrow and adipose tissue derived MSC EVs. This study focuses on elucidating the immunomodulatory properties of EVs derived from DPSCs. DPSCs are mesenchymal stem cells that reside in the dental pulp. With dental caries (enamel and dentin degradation and dental pulp infection from bacterial invasion) being the most prevalent infectious disease in both adults and children, the dental pulp cells have evolutionarily and anatomically evolved to offer protection and defense.

Currently, there is considerable published literature on the regenerative properties of dental tissue derived EVs (includes all dental sources such as periodontal ligament, apical papilla, pulp and gingiva) that highlight their potential for bone, dental pulp, periodontal tissue and nerve regeneration (23, 45–47). However, from an immunomodulatory perspective, the role of DPSC-EVs is severely understudied. From a translatory perspective, DPSCs possess several advantages over other MSCs in that, they can be isolated from adults during routine dental procedures and more easily and less invasively than bone marrow MSCs from the deciduous teeth of children (48). This, combined with their enhanced stability under cryopreservation (49, 50) makes them an attractive source for MSC EVs. One recent *in vitro* study compared the effects of bone marrow and dental pulp MSC derived EVs on CD4+ cells and identified that DPSC-EVs showed enhanced immunomodulatory effects on these cells compared to bone marrow MSC EVs (51). This was an interesting observation and the mechanistic details of this function remain as critical knowledge gaps.

In this manuscript, we have focused on evaluating the ability of DPSC-EVs to influence macrophage polarization under inflammatory and reparative scenarios. Initial comparisons with bone marrow MSC derived EVs confirmed the enhanced properties of DPSC EVs. We also noticed that DPSC-EVs had a more specific effect on iNOS and TNFα expression that was significantly different from that of bone marrow MSC EVs. In prior studies, we had identified that MSC EVs’ immunomodulatory function is enhanced when preconditioned with inflammatory cytokines such as TNFα (31). Considering the fact that DPSCs are anatomically primed to react to bacterial infection, we evaluated if LPS preconditioning of DPSCs will alter its immunomodulatory properties.

Our results showed that there in an increase in the anti-inflammatory property of DPSC-EVs with LPS pre-conditioning with both gene and protein expression studies showing reduction in pro-inflammatory marker expression. With respect to the pro-reparative activity of the EVs, the LPS pre-conditioned DPSC-EVs showed a remarkable ability to enhance reparative macrophage phenotypic polarization. We then analyzed the expression of various phospho proteins involved in the NFκb pathway and observed that both naïve and LPS pre-conditioned DPSC-EVs significantly reduced the phosphorylation of these proteins and thereby, negatively regulating the pro-inflammatory pathway-specific activity in primary mouse macrophages. Collectively, these results underlined the ability of the DPSC-EVs to negatively impact the pro-inflammatory polarization of macrophages and positively impact reparative polarization.

The *in vitro* experiments were performed by polarizing macrophages with pro inflammatory and pro reparative cytokine stimulation. While such experiments serve well in identifying molecular mechanisms of action, the observations under these conditions do not reflect *in vivo* scenarios accurately. Therefore, we evaluated the properties of naïve and LPS preconditioned DPSC-EVs in an injury induced inflammation model. For this purpose, we utilized a calvarial bone injury model and observed the effects of the EVs over a period of five days. This injury model creates acute injury related inflammation and serves as a good testing platform to study the anti-inflammatory activity of the EVs. From prior studies using this model, we were aware that inflammation resolves naturally in 7 days. Here, we wanted to observe if this would be accelerated in the presence of the EVs.

Our results indicated that both naïve and LPS preconditioned DPSC-EVs reduced the presence of iNOS positive cells at all time points and increased the presence of ARG1 positive cells at days 1 and 3 with the system seemingly returning to basal levels by day 5 indicating positive resolution of inflammation. Conversely, the control group showed robust inflammation as expected with a gradual increase in the presence of ARG1 positive cells with time. The increased presence of ARG1 positive cells at day 5 in the control group serves as an indicator that the resolution of inflammation in this group was lagging behind the other EV treated groups. We further examined the effects on TNFα and TRAF6 by immunohistochemistry and observed that similar to our *in vitro* observations, both naïve and LPS preconditioned DPSC-EVs reduced their expression with LPS DPSC-EVs showing more robust activity with respect to reduction of TRAF6 expression. Collectively, these results confirmed our *in vitro* observations and highlight the anti-inflammatory activity of the DPSC-EVs from both an acute inflammation perspective (animal model) as well as from the perspective of LPS inflamed macrophages (*in vitro*).

In prior studies with MSC EVs, us and others have highlighted the role of EV resident miRNA cargo (31, 32, 42). Here, we compared the top 10 most expressed miRNA from bone marrow derived MSC EVs from our earlier study with that of DPSC-EVs and noted that miR-320a-3p was unique to DPSC-EVs and that its expression was increased upon LPS preconditioning of DPSCs. We restricted our comparison to the top 10 miRNAs alone this list encompasses greater than 70% of the entire EV miRNA composition. Prior published studies have shown that miR-320a-3p negatively regulates the NFκb pathway (52–55) as well as promotes reparative macrophage polarization (56–58). Based on these facts, we hypothesized that this miRNA could play a role in the unique activity of DPSC-EVs on this pathway. Using a series of mimics and EV activity experiments, we evaluated the functionality of this miRNA. Our results did not conclusively prove that this miRNA is the only factor in DPSC-EV activity. However, results showed clearly that this miRNA may play a prominent role in DPSC- EV function with combinatory experiments with both mimics and DPSC-EVs behaving similar to LPS preconditioned DPSC-EVs by enhancing the anti-inflammatory activity compared to naïve DPSC-EVs alone albeit minimally.

In this context, it is to be noted that miRNA activity in different cell types is dependent on the abundance of its target genes as well as the stoichiometry and kinetics on interaction with the target. From this perspective, while we can attribute some of the activity of DPSC-EVs on the NFκb pathway to this miRNA, the mimic experiments may also include activity of the miRNA on other pathways. Additionally, the enhanced effects of the EVs compared to the mimics could also be equated to other EV components such as its protein, mRNA and lipid composition. Further studies with lipidomics, proteomics and mRNA sequencing are required to elucidate and conclusively identify individual roles of defining contributors.

Overall, the results from this study highlighted the immunomodulatory properties of DPSC-EVs and its effects on the NFκb pathway. Our results explain the increased immunomodulatory activity of the DPSC-EVs compared to that of their bone marrow counterparts and identify miR-320a-3p as a possible significant contributor to this uniqueness. From a therapeutic perspective, this study shows the potential of naïve DPSC-EVs as agents to reduce site-specific inflammation. The sequencing results and the identification of miR-320a-3p provide valuable clues to pursue miRNA-based EV engineering in future studies by us and others.

## Data Availability

The datasets presented in this study can be found in online repositories. The names of the repository/repositories and accession number(s) can be found below: PRJNA1123286 (SRA).

## Supplementary material

Supplementary 1 EVs from regular DPSC and hBMSC cells were sequenced, top miRNAs from both EVs were graphed and these showed the higher expression of miRNAs in DPSC as compared to hBMSC and some specific DPSC miRNAs (miR 320a, 127 and 199a).

Supplementary 2 Whole membrane images of immunoblots blots depicted in **Figure 5**.
