# Supplementary figures and images for: Immunomodulatory properties of naïve and inflammation-informed dental pulp stem cell derived extracellular vesicles
Source: Front Immunol. 2024 Aug 19;15:1447536. doi: 10.3389/fimmu.2024.1447536 (PMC11366660; doi:10.3389/fimmu.2024.1447536)

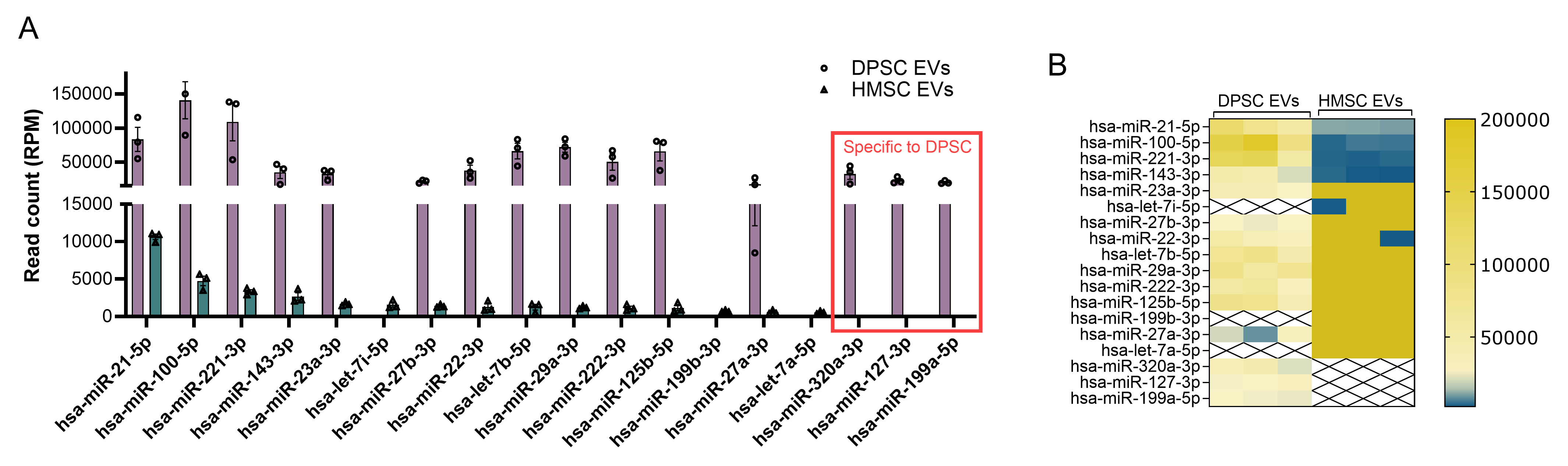

Supplement: Supplementary 1 — EVs from regular DPSC and hBMSC cells were sequenced, top miRNAs from both EVs were graphed and these showed the higher expression of miRNAs in DPSC as compared to hBMSC and some specific DPSC miRNAs (miR 320a, 127 and 199a). [file Image1.tif]

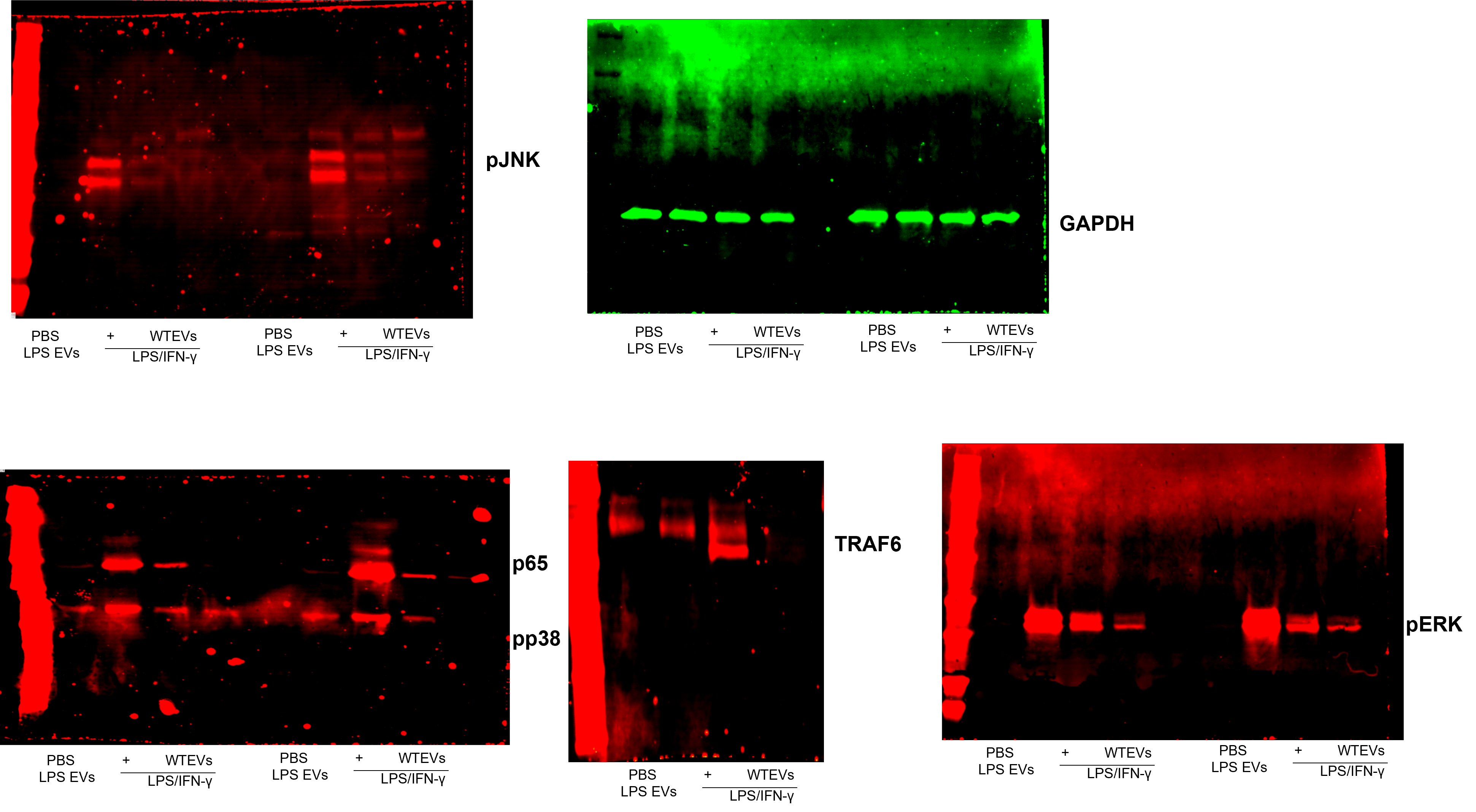

Supplement: Supplementary 2 — Whole membrane images of immunoblots blots depicted in Figure 5 . [file Image2.tif]
